# Coping efforts and resilience among adult children who grew up with a parent with young-onset dementia: a qualitative follow-up study

**DOI:** 10.3402/qhw.v11.30535

**Published:** 2016-04-08

**Authors:** Aud Johannessen, Knut Engedal, Kirsten Thorsen

**Affiliations:** 1 Norwegian National Advisory Unit on Ageing and Health, Vestfold Hospital Trust, Tønsberg, Norway; 2 Norwegian Social Research NOVA, University College of Oslo and Akershus, Oslo, Norway

**Keywords:** Adult children, coping, early-onset dementia, experiences, longitudinal qualitative study, resilience, services, support

## Abstract

**Background:**

It is estimated that one in four persons with young-onset dementia (YOD) (<65 years old) has children younger than 18 years old at the onset of the dementia. These children experience a childhood different from what is expected. Adult children of parents with YOD are seldom addressed in research, and the impact of the dementia on the children's development over time has rarely been studied.

**Aim:**

The goal of this study was to explore how adult children experienced the influence of their parents’ dementia on their own development during adolescence; what coping efforts, strategies, and resources they employed; and how they evaluated the most recent changes in their life situation.

**Method:**

A follow-up, grounded theory approach in two phases was used. Qualitative interviews with 14 informants (18–30 years of age) were conducted in 2014 and one year later, in 2015.

**Findings:**

Nearly all the informants expressed that their emotional well-being and their life situation were better at the second interview compared to the time of dementia onset in their parents. To overcome the difficulties of being a child of a parent with YOD, they used different instrumental, cognitive, and emotional coping strategies, subsumed analytically under the concept *detachment*. This category covers three subcategories of coping strategies: *moving apart*, *greater personal distance*, and *calmer emotional reactions*. Another category, *resilience*, designates combinations of the coping strategies. Vital for the development of coping resources and resilience was the need the informants had for social support—for people they saw who listened to them and responded to their needs.

**Conclusion:**

Most of the informants reported that they experienced a better life situation and less emotional stress over time as their parent's dementia progressed. They developed better coping capacities and greater resilience. Vital for the development of coping resources and resilience was the need the informants had for social support.

Being an adolescent, struggling to develop self-confidence and coping capacities to establish oneself as a well-functioning adult, is a demanding and complex task for all youngsters. The young person is in a transitional state—moving towards “emerging adulthood”—where she or he is expected to explore and establish her- or himself in important arenas of life: education, work, family life, and parental roles (Arnett, 2004). To mature cognitively and emotionally and gradually master tasks necessary for independent life, children need support from parents who are reasonably well functioning in their parental roles.

To grow up as the child of a parent with young-onset dementia (YOD) is to be deprived of such support from one parent and to be exposed to obscure signs and changes in the parent's reactions by an unexpected and unknown disease, resulting in a transformed parent–child relationship. The stress of this situation influences the child's development and everyday life (Allen, Oyebode & Allen, 2009; Barca, Thorsen, Engedal, & Johannessen, 2014; Gelman & Greer, 2011; Hutchinson, Roberts, Kurrie, & Daly, 2014; Johannessen, Engedal, & Thorsen, 2015).

A person under 65 years old who is diagnosed with a dementia syndrome is often referred to as having YOD. Compared to the 78,000 people in Norway who are above the age of 65 and have dementia, the number of people with YOD in Norway is estimated to be 3,000 (Engedal & Haugen, 2009; Prince et al., 2013).

It is estimated that at least one in four persons with YOD has children younger than 18 years at the onset of dementia (Haugen, 2012). Most dementia disorders are of neurodegenerative origin and progress gradually. Therefore, the family might encounter increasing challenges over time. Psychological symptoms of dementia and the occurrence of frontotemporal dementia are more common among people with YOD than in the older population with dementia (Van Vliet, 2012). Thus, the behaviour of the person with YOD may be challenging to the family. Because of cognitive and behavioural changes in people with YOD, their children (in different age groups) will easily become worried, confused, and fearful when important information is forgotten and daily routines are broken (Allen et al., 2009; Barca et al., 2014; Johannessen et al., 2015; Kjällman-Alm, Norbergh, & Hellzen, 2013).

Further, the children may experience shame and feel embarrassed when their friends meet their parent who is forgetful or exhibits a surprising personality (Allen et al., 2009; Johannessen et al., 2015). As a result, the children often retract from contact with friends and find it difficult to confide in friends about their home situation. It is challenging to live a normal adolescent life and perform at the same level in school and at work as before when living with a parent with dementia. It is not unusual for conflicts with the parent and other family members to develop (Allen et al., 2009; Barca et al., 2014; Johannessen et al., 2015).

Earlier studies have pointed out that it is important for adult children of parents with YOD to be given opportunities to express their thoughts, feelings, and perceptions of their present needs and situation (Allen et al., 2009; Barca et al., 2014; Johannessen et al., 2015; Millenaar et al., 2014; Svanberg, Stott, & Spector, 2011).

*Children* is a term with different meanings. *Childhood* is a stage in the life course, often seen in contrast to *adulthood*. Life stages in the first part of life have been further differentiated in Western industrialized countries as childhood, adolescence, and, as recently termed, *emerging adulthood*, for young people 18–25 years old (Arnett, 2004). The terms depend on historical, geographical, and cultural contexts (Elder, 1979; Elder & O'Rand, 1995; Giele & Elder, 1998, pp. 457–475) and vary among research fields. With regard to family generations, people are *children* of their parents even if they may be 80 years old and their parents are centenarian (Hagestad, 1990). In this article, the informants, between 18 and 30 years of age, are referred to as *adult children*. When they retrospectively discuss their earlier life stages, we use the terms *children* or *adolescents* and use *childhood* (children) as a life stage contrasted to the adult stage.

## Theoretical background

### Development in adolescence: coping and resilience

To develop a robust sense of self, identity, self-respect, and self-confidence and to become a vital actor in one's own life are the main tasks of youth (Erikson, 1959, 1968). The parents are mirrors for their children (Kohut, 1971, 1985), meaning that they are models for their children in this process. Children are dependent on support from well-functioning, loving, and supportive parents to develop and establish a robust identity and sense of self with adequate coping resources (Bosma & Gelsma, 2003). A parent–child relationship with a parent with dementia deviates from what is considered a normal relationship (Gelman & Greer, 2011; Kindermann, 2003; Stratton, 2003). Growing up with a parent with YOD implies stress and burden (Allen et al., 2009; Barca et al., 2014; Johannessen et al., 2015; Kjällman-Alm et al., 2013).

Coping has been shown to modify the impact of stressful and taxing situations and living conditions. A classic definition of coping is the one from Lazarus and Folkman (1984, p. 141): “Coping is the constantly changing cognitive and behavioural efforts to manage external and/or internal demands that are appraised as taxing or exceeding the resources of the person.” *Coping*, in their theoretical perspective, refers to the *processes* of coping, underlining efforts to manage, irrespective of the *results* of the efforts. Managing can include minimizing, avoiding, tolerating, and accepting the stressful conditions, as well as attempts to master the environment (Lazarus & Folkman, 1984, p. 141). The concept of *coping* is used in this study instead of *mastering*, which denotes (more) successful results. Lazarus and Folkman (1984) emphasize that coping strategies takes place within specific contexts that efforts are directed towards particular conditions and that different coping strategies may be combined. Coping strategies can both facilitate and impede each other, and they are complex and context-dependent.

Lazarus and Folkman (1984) concentrate on two main strategies to handle stress and adversities: *problem-focused* (instrumental) coping and *emotion-focused* coping. Problem-focused coping aims at altering the stressful circumstances by actions. Emotion-focused coping changes the emotional responses to the problem; this strategy is often used when one lacks control over the most significant aspects of the situation. People use *cognitive appraisal* to evaluate a situation and consider coping alternatives. Cognitive reappraisals may also change the meaning of the situation, without changing it objectively; this is a cognitive form of emotion-focused coping (Lazarus & Folkman, 1984, p. 150).

An important aspect of being able to handle stress well is an accumulated feeling of being able to *be in control*, to have the necessary resources to handle and change the taxing situation in a favourable way, and to reduce stress (Daatland & Solem, 2011; Slagsvold & Sørensen, 2013). A major distinction is often made between control aimed at changing the external environment and control targeted towards internal mental processes, as in Heckhausen and Shulz's (1995) concepts *primary* and *secondary control*. Concepts referring to control usually describe a person's *sense of being in control*, irrespective of whether the person has control empirically (Slagsvold & Sørensen, 2013). Having a sense of being in control (*perceived control*) is associated with multiple positive aspects of life, such as a positive quality of life, well-being (Ferguson & Goodwin, 2010; Lachman & Agrigoroaei, 2010), and physical and mental health (Bjørkløf, Engedal, Selbæk, Kouwenhoven, & Helvik, 2013; Ward, 2013).

The concept *resilience* denotes the ability to withstand stress and develop mastering resources, despite hardships. There is no single definition of resilience, but the concept usually entails three aspects: psychological and dispositional attributes, family support and cohesion, and external support systems (Masten et al., 1999). Masten et al. (1999) found high-quality parenting to be a significantly protective factor contributing to resilience in children. Other studies have documented parents’ influence in achieving a longer lifespan and for supporting the development of resilience in children with stigmatizing disabilities (Schanke & Thorsen, 2014).

The adult children of parents with YOD have often been seen as carers, as in studies concentrating on examining and measuring caregiver stress and burden (Svanberg et al., 2011). The consequences for adult children's development while growing up have been outside the scope of previous studies. To our knowledge, no longitudinal qualitative studies focusing on adult children's coping efforts to live with a parent with YOD and on how the situation influenced their development during adolescence have been carried out. Thus, the aim of the present study was to explore how adult children experienced the influence of their parents’ dementia on their own development during adolescence; what coping efforts, strategies, and resources they have employed; and how they evaluate the most recent changes in their life situations.

## Methods

### Design

The study was an explorative, descriptive study with the aim of gaining new knowledge about phenomena that have been minimally researched: the development over time of the children of parents with YOD. Corbin and Strauss (2008) outlined a reformulated approach to grounded theory. The reformulated method (see the section “Analysis”) is particularly suitable for the study of people's lives through processes and social interactions, making it appropriate as the chosen method for this study. We have applied the method described by Corbin and Strauss (2008) without the intention of formulating a theory but rather to give more precise insight into a rather uncharted field. Their methodological approach is seen as fruitful in acquiring knowledge about how young adults perceive the influences of their parents’ dementia on their own development during adolescence, what coping efforts they have employed over time, and how they evaluate the most recent changes in their life situations. In the families we studied, several processes and interactions took place in complex ways: The children's interactions with their parents and families developed while the parent's dementia was developing, and family relationships were transformed.

A follow-up design with two interviews performed 1 year apart was applied in order to capture experiences, coping efforts, and strategies over time. Both interviews covered experiences today as well as retrospective accounts of earlier reactions. A short time period was chosen to get detailed and more precise information about changes taking place, while the experiences and reflections were “fresh.”

#### Participants

To attain heterogeneity, we included adult children, 18 years of age and above, from the southern and western parts of Norway. They were recruited from two memory clinics, one municipality dementia team, a nursing home (NH) for persons with YOD, and from the Norwegian National Support Group for Adult Children.

A total of 16 adult children were asked to participate, and two declined. Thus, the sample at the time of inclusion (at the first interview) consisted of 14 informants, aged 18–30 years of age (mean 24 years). The mean age of the parents was 61 years. The characteristics of the informants and their parents are described in Table I. The diagnoses were made 6 months to 10 years before the interviews took place.

**Table I:** Characteristics of adult children and parents with YOD at the first and second appointments.

Adult child					Parent with YOD		
	
		Household				Household	
					
Relationship (age)	Siblings	Appointment 1	Appointment 2	Relationship (age)	Civil status	Appointment 1	Appointment 2
Daughter (18)	Two half siblings^a^	Parental home^b^	Alone	Father (63)	Married	Home	Home
Daughter (26)	Two siblings	Alone^b^,^c^	Partner	Mother (59)	Widowed	Nursing home	Nursing home
Daughter (26)	Five siblings	Alone^b^,^c^	Dropped out	Mother (66)	Divorced	Nursing home	
Daughter (30)	Two siblings	Partner^c^	Partner	Mother (59)	Widowed	Nursing home	Nursing home
Daughter (27)	Two siblings	Spouse^c^	Spouse	Mother (65)	Married	Home	Home
Daughter (24)	None	Partner^b^	Partner	Mother (62)	Divorced	Nursing home	Nursing home
Daughter (26)	One sibling	Partner^c^	Partner	Father (61)	Married	Home	Nursing home
Daughter (29)	One sibling	Spouse^c^	Spouse	Father (62)	Divorced	Home	Nursing home
Daughter (27)	One sibling	Partner^b^,^c^	Partner	Father (63)	Married	Home	Home
Son (20)	Three siblings^a^	Parental home^b^	Alone	Father (60)	Married	Home	Nursing home (partly)
Son (18)	One sibling	Parental home	Parental home	Father (61)	Divorced	Nursing home	Nursing home
Son (19)	Three siblings^a^	His sister's family^b^	His sister's family	Father (62)	Married	Home	Home
Son (26)	One sibling, two half siblings^a^	Partner	Partner	Father (57)	Married^d^	Home	Home
Son (20)	None	Alone^b^	Alone	Father (60)	Married	Home	Home

YOD, young-onset dementia.

a Do not live together in the same household.

b Lived together with the parent when the first signs of dementia were revealed or the diagnosis was made.

c Have their own children.

d The parent married again.

#### The two interview phases

Individual qualitative interviews were conducted in two phases: Phase 1, at inclusion (2014), and Phase 2, one year later (2015). This rather short period was chosen because it was possible that significant aspects of both the adult children's and the parents’ situation would change rapidly. Dementia patients can in some cases deteriorate very fast, and the young people's life situations might have changed over a short time period as well (Arnett, 2004). The young people might have moved out of the parental home, started studying, started a working career, or established their own families. The interviews, conducted by the first author (AJ), were performed at the most appropriate place for the informants (Denzin & Lincoln, 2011; Kvale, 1983, 1989) and were audio recorded. Within 2 weeks, they were transcribed verbatim by a professional typist. A quality control check was performed by the interviewer, who listened to the tapes while reading the interviews.

Both interviews were based on the same guide with six broad, open-ended thematic questions focusing on the informants’ experiences of having a parent with YOD. The questions are listed in Table II. Depending on their replies, the aspects and ideas raised by the informants led to further questions to obtain additional information.

**Table II:** Questions and themes in the interviews of the adult children.

How was it during those years and how is it now for you to have a parent with dementia? How has this disorder affected your life in the different stages of the disorder? How has the disorder affected your family, the relationships within the family, and among your friends in the different stages of the disorder? When you look back, is it possible for you to describe something that has been or could have been of help to you or your family during the different stages of the disorder? What kind of support have you received or do you receive, and what are you in need of today? Have you had to make decisions for your parent, and if so how has that been?

#### Phase 1

Nine of the fourteen interviews took place at the informants’ homes, three at the interviewer's workplace, and two at the informants’ workplaces. The interviews lasted for 15–51 min (mean 39 min).

#### Phase 2

One daughter declined participation at the follow-up. Two of the adult children had moved out of their parental homes since the first interview, one parent had moved to an NH, and another parent was living partly in a NH. Eight of the interviews took place at the informants’ homes, four at the interviewer's workplace, and one interview took place at the informant's workplace. They lasted for 14–67 min (mean 32 min). In addition to the questions on the interview guide, detailed follow-up questions explored more specifically what had happened since the first interview. The informants were posed a summarizing open question: “All in all, how have you experienced the development of your situation since we talked together the last time?”

### Analysis

The transcribed interviews were analysed with a focus on the adult children's experiences and efforts to cope over the period of time as the child of a person with YOD. The analysis was performed by two of the authors in line with the modified version of analysis described by Corbin and Strauss (2008). The initial analytical approach (open coding) was to read all the interviews open-mindedly for relevant codes. Then the codes were compared to find the most relevant higher level codes (axial coding) for the whole group. At the same time, subcategories and variations were noted. A re-reading of the interviews showed that narratives about emotional reactions, cognitive coping, and efforts through actions were usually related to interactions and acquired their meaning within social relations. Agreement on higher level codes was reached by discussions and reflections among the researchers. During the process, the researchers continuously related the codes to the empirical material, analysing it *vertically* and going back and forth in the material of one informant. We also analysed it *horizontally*, comparing the informants on different themes, such as family relations, in line with the method's constant comparative approach. As Corbin and Strauss emphasized (2008, p. 198): “Open coding and axial coding go hand in hand.” Empirically, the distinctions are for explanatory purposes.

The time perspective was from reported changes between the first and second interview. We examined experiences, emotions, and events that differed over time, noted the strategies used, and made evaluations of the outcomes. Retrospective information was collected at both the first and second interviews—in that aspect there were no principal differences, as Plumridge and Thomson (2003) underlined. The narratives of change cover a longer period of time in the first interview. The memories are less precise and influenced by later development. As noted, the second interview was performed to grasp more clearly the experiences of change within a short and definite period of time and to see how reactions were attributed to the changes.

### To attain credibility and trustworthiness

During every stage of the study, we focused on securing what Lincoln and Guba (1990) called a “credibility” and “trustworthiness” of the findings. The interviewer posed new questions during the interview to obtain more information (Kvale & Brinkmann, 2010). As Kvale (1989) emphasized: “To validate is to question.” The transcriptions were controlled by the interviewer, who listened to the tapes repeatedly to ensure that they were correct. Significant excerpts from the interviews were compared and analysed, and the most representative excerpts were eventually included in the text under higher order themes. We adhered to the criteria of Corbin and Strauss (2008, p. 300)—that sufficient detail and description should be presented so that the readers feel that they can judge for themselves. During the analysis, the researchers repeatedly discussed the codes and themes. We also focused on the criteria posed by Charmaz (2006, pp. 182–183) for evaluating credibility: establishing logical links between the gathered data and the argument and analysis.

### Ethics

The study followed the ethics outlined in the revised Declaration of Helsinki (World Medical Association, 2013) and was approved by the Regional Committee for Ethics in Medical Research, Southern Norway (2013/2149). The Norwegian Data Protection Authority also approved the study (36797). The informants received oral and written information about the study and gave written consent before they were interviewed.

## Findings

The dominant desires and strategies in the coping efforts of the adult children were captured by our determination of the main category, *detachment*, which includes several approaches as behavioural actions, cognitive strivings, and emotional adaptations that are related to “outer events.” The coping approaches may take place separately, but they are often interrelated at different stages, influencing and modifying each other. The coping efforts take place while the child is maturing and the parent's dementia is progressing. We examined the various aspects of the detaching approaches presented in this sequence: behavioural actions, cognitive strivings, and emotional adaptations. Moreover, we looked at the approaches to see how these may combine to become resilience. The analysis resulted in three subcategories, denoting the main aspects of detaching: *moving apart*, *greater personal distance*, and *calmer emotional reactions*. In addition, another category, *resilience*, was analysed as the combining of coping strategies, resulting in better coping resources. The coping efforts were contextualized. The outcome was often felt as relief at the time of the second interview—the situation had become “better.” The informants felt that they were more able to handle the challenges and go on with their own lives.

### Moving apart

In the subcategory of *moving apart*, most of the adult children and the parents with dementia had moved out of the family home, resulting in a totally changed living situation that influenced their relationship. A young man who recently moved out of the family home described his experiences: “I had thought of it earlier, but I took the decision while we were at the gathering (for children—adolescents—of parents with YOD).” Then I said to Mum: “Now I have been Daddy to my dad for eight years. That is enough! Now it is time to move out, so that Erik can be Erik—be young at last.

He gave the following reasons for his feelings of relief: “It has been wonderful not being so close to my father all the time. There has been so much unnecessary quarrelling, and it is okay to escape from it, since I—in spite of everything—am *the son* of my father. This is what I will be, not the father of my father.” He decided and acted accordingly, to change the life situation and the role he was playing in his father's life. He could be *young*, able to act individually, and take his life to the next stage—emerging adulthood.

Another person who had moved out also experienced the changes with great satisfaction. She summarized the change with these words: “Staying at home was dreadful. Moving out was wonderful. It is much better to live away from my mother and father.”

Both informants stated that their relationship to the parent with dementia had become better after moving out. They now visited the parent when they feel in “a good mood,” and the relationship had improved.

The two informants were now able to concentrate on other things in life. They described many valued aspects of their new lives outside the family home. Moving away had reduced the daily conflicts and caretaking tasks, the stress of always being tense and alert for the parent's reactions. They valued being able to retreat, having time for themselves, and not seeing all the details all the time of the parent's state. One of them said, “It hurts to see it.” The male informant mentioned an important aspect for him—to escape the task of having to handle intimate care in critical situations: “It was too unpleasant for both of us that I had to monitor that he was clean and not wet. Now it is out of the question.”

The young man used the word *distancing* when summing up the situation: “It is better because I left home and have distanced myself more. I can retreat. This is *my* place! Here is *me*!” Now he was the centre of his own life. He was becoming an *I* and was acting on his own behalf. The woman emphasized, “Now I am not the parent of my parent. That is what I was. Now I am in control of my own life.”

The informants had often moved out of the family home in steps. They had started on programmes of study, had gone abroad, or had gotten a new job, but they often came home for both shorter and longer periods until they moved out permanently. They adapted gradually to greater physical distance. In addition, many of the parents with dementia had moved out of the family home. At the time of the second interview, seven parents lived in an NH after attending a day care program or had periods of respite care in an NH. This break in informal care was welcomed as a great relief. A male informant described the feelings he had when his father was in respite care in an NH: “I felt it when I lived at home when my father was in respite care: When you sat down on the sofa, you actually relaxed. You really relax! It is a relief, really. You sit there and feel how quiet it is in the house: For usually you go there tense, waiting for something to happen.”

### Greater personal distance

Within the subcategory of *greater personal distance*, detachment was a gradual and multifaceted development. Realizing that distance to the parent was part of the personal development process for most of the informants. A married woman with a baby described her relationship to her father, who had recently moved into an NH, like this: “I have distanced myself from everything concerning my father much more than before. Last time when we talked, I was much more in the middle of the process, when there were things to do and handle.” Physical distance seemed to increase the experience of personal detachment, but it could also have been the result of more active decision making.

Personal detachment may be the outcome of recognizing a former personal distance: “My brother and I recognized that he had not prioritized us when we were growing up. Then the reaction came: Why should we prioritize him when we have our own families that we would like to spend more time with? I have put it more to the side, can you say, I prioritize my daughter. I do that. So sometimes he is forgotten during everyday life, the daily chores engulf you!” For the informants who had established their own families, these families came first. In the stage of emerging adulthood, many tasks and obligations require attention. Daily life is often exhausting, and fewer responsibilities and caretaking tasks for the parent are welcomed.

Cognitive distance also increases with the progression of dementia. In the first and second interviews, the parents were described as becoming more remote, disinterested in the child, and not “like themselves.” The parent “disappears into the fog,” as one informant described it. The informants were less able to recognize and “reach” the person, the father or mother as they knew them. One woman narrated the changes she sees in her mother: “The person she was, I see her like that in short moments, but at the same time far from that. Only glimpses. But she dwindles away.” The gulf widens between parent and child. A young man reported: “I have nothing left of my daddy.”

Acceptance of the diagnosis also takes a long time. The fact that the personality changes are the result of a disease, not forgetfulness, neglect, or a difficult personality, has to be contemplated, experienced, and absorbed gradually. One informant said, “At a meeting with other children (adolescents) of parents with YOD, we sat and talked about memories of the time before the disease. It is very difficult to remember how it was before the illness starts. What I think is that after Daddy is dead and I have finished that period of my life and have sorted things out a little bit, that the memories gradually and slowly will return. We have a lot of pictures that I have not dared to look at yet. I think I will wait a while with that, before I sit down and look at pictures from my childhood. I think that it will break me down a little.”

Here we see the young people discussing both their collective experience of early memories being “closed off” and their deliberate use of strategies to keep remembrance about the happier times of childhood out of mind. Cognitive distancing, thus, may occur in many ways: by acceptance of the diagnosis, by recognition of the changed personality of the parent, by becoming more personally remote, by mental efforts to avoid negative interfering thoughts, by living apart from the parent, and by engagement in other arenas of life.

The respondent with the most rational, unemotional narrative of his experiences had had 10 years of experience since his father had been diagnosed with dementia. He was then 10 years old. He said: “When you got to know the reason why things were like they were, then it was much easier to accept it as it was. The quicker you accept the realities, the easier it is to move along. I think it is something I thought of already 10 years ago. It is really to accept it the way it is and then make a course for where to move from there. All this is outside our control, and I cannot let this restrain me from doing what I have to.” Here, he quickly shut away any feelings of guilt about living abroad because it was not “at all constructive,” so “it bothers me very little.” He did not find the situation difficult at all; it was the way it is. It had become “normalized.”

One informant did not experience his situation as better by the time of the second interview; it was “just the same.” For many years, he had had a distant relationship with his father, who had been divorced and lived with his second wife and their children.

### Calmer emotional reactions

In the subcategory of *calmer emotional reactions*, emotional stress seemed to have lessened by the time of the second interview, and most of the informants’ feelings had changed since the first interview. The woman who recognized that her father had never prioritized her told us, “I am at a completely different place now. I am that! Especially, emotionally, both concerning care for daddy, and a feeling of guilt. Yes! The feeling of guilt has disappeared, and that is wonderful. It is this feeling that has been the most burdensome.”

All the interviews were filled with narratives of emotions, but the intensity of the emotions seems to have lessened from the time of the first interview to the second. The first interviews were often filled with stories about *anger*. This feeling was often directed at “fate”—this incomprehensible disease distorting their youth and futures. However, “fate” also referred to conflicts with the parent and other family members. By the second interview, many informants seemed to have moved to a calmer emotional state, and their narratives showed more acceptance. One informant expressed, “The shock of the diagnosis is over; we have in a way accepted that it is the way it is.” The informants reported feelings of sorrow for their parent with dementia. They pitied him or her, regretted that they had lost personal contact with their parent, and missed “the person” as well as the parental role. They felt sad.

The feeling of *guilt* was mentioned. One informant said, “Sometimes I choose not to visit her, because then my whole day is spoiled. But then you have to go, or the feeling of guilt is even worse.” Some received the advice to not feel guilty for their visiting pattern. One informant said, “They often mentioned [at the group meeting] that I should not feel guilty for not seeing him so often.” Some described exhausting emotions when they visited their parent at the NH: “I try to distance myself, but get caught up when I see my mother. I get a very bad feeling inside when I see her. I dread seeing her, and then it goes badly. I feel unwell for days afterward or sad and depressed.” Her efforts to distance herself emotionally were in vain for several days, until the feelings calmed down.

Others mentioned that they feared the time to come, to see the dementia progressing into the last stages and then into prolonged dying. One woman described her feelings: “We are watching her die slowly in a way, and that grief is prolonged over a long time. It is like sitting at her deathbed in a way. We can think of the time when it is over, and we really can mourn.” Some try to cheer up their parents. “I try to be strong when I visit her, to smile, joke, have fun together. We dance to Elvis, eat ice cream and goodies. Have a nice time!”

Humour is mentioned as an outlet, a strategy for taking the sting out of the sadness and helping the participants to handle the situation. It gives emotional relief. It is shared sometimes with the parent with dementia, but laughter and joking are valued also in other relations. “My best advice to all those new to this situation is: use a lot of humour! You have much to gain!” said a young man.

### Resilience

Over time, the respondents developed better coping abilities—resilience. The road to resilience varied. It was often complex; the respondents combined various strategies and adapted to different circumstances. One vital factor seemed to be social relationships—meeting other people who could understand and support them. The analysis revealed that even one single event could transform the situation completely—like meeting one person the respondents could confide in and get support from. One woman described a decisive event: “I met a man just before you came to interview me the first time. It has become better the last year. Now I have one who always is there, always supports me, and joins me when I visit mammy. Mum loves him. Earlier I had no one. I was completely alone.”

Another important social experience was meeting other young people who were in the same situation, who had a parent with dementia. The respondents said that suddenly they did not feel completely alone. In particular, those participating in weekend meetings with other adolescents described the meetings as “a revelation,” an “aha” experience. “The meetings are fantastic! I have got friends that I can talk to. Now I know that there is someone in the same situation as me living close by. It was wonderful to ‘empty’ myself and be understood. Yes, unbelievably nice. I have other friends, but they do not understand it in the same way, they just understand it like they say: ‘Yes, my grandmother also has it.’ Now I am … I do not feel so alone.”

At the meetings, the participants would talk “about something very sad, and do something fun afterwards. Then it is much easier to talk about it.” Discovering that they were not alone, communicating in a setting where they could talk openly about problems, and also sharing enjoyment seem to have been the key to the meetings’ significance. Some advice from one enthusiastic participant to others: “Accept all the offers of help you can get, even if it feels extremely difficult. So, just jump over that obstacle, because then it becomes much better. And be open about it!” However, some informants did not think that such meetings were reflective of their situations.

In most other cases, the resilience that resulted from several interrelated events and coping efforts also led to greater acceptance of the disease. One young man who had recently moved out of his parents’ home summarized his situation by pointing at several factors that improved his situation: “My life is better now because a year ago I had not really understood it. I thought: It is not Daddy who has dementia! But now I have accepted that he has the disease. I have got friends in the same situation as me, and I have moved out—that makes it easier to get in touch with him. I feel it is better. I really do! I would not feel that way if I still lived at home. I feel that I have it as good as possible, considering that he is ill.” He has also found a new full-time job, which he is very happy about. He has an understanding boss who is flexible with the informant's sleeping problems and stressful situation with the parent. The situation now is, as he summarized it, “as good as possible.” He aptly describes the different changes—“outer” and “inner”—as interrelated. They are transforming his life in positive ways, and now he is handling the situation much better. The outcome is resilience and better coping, more maturity, and higher life satisfaction. Thus, we find that the development of resilience is contextualized, often in multifaceted and complex ways. However, it can also be primarily the result of one event or one personal relationship. Resilience may be more or less robust, or vulnerable. Social support is vital.

All in all, the interviews at both phases showed that, in spite of some differences in the respondents’ observations, almost all of them believed that their emotional well-being and life situations were better today compared to the time of dementia onset in their parents. Those who had accepted the situation, distanced themselves more from the parent with dementia, and obtained support from others expressed better emotional well-being. The informants admitted that life still had ups and downs. In particular, those adult children who still worried about the care given to their parent had down periods. The feelings of loss of the parent were still there, along with some of the feelings of guilt, but altogether life had become better.

## Discussion

Consistent with other studies, we found that children growing up with parents with YOD experience great stress and emotional burden (Barca et al., 2014; Gelman & Greer, 2011; Johannessen et al., 2015; Millenaar et al., 2014). These children pass into adulthood with a parent–child relationship that radically deviates from the expectations of a “normal” childhood (Gelman & Greer, 2011).

However, the adult children in our study learned different ways to master aspects of their situation as time went by, and their everyday lives improved significantly over the year between interviews. The informants’ descriptions of coping efforts varied, were mainly contextualized, and related to situational factors. The coping strategies were adapted to whether the respondents had previously lived or still lived with the parent with dementia, what their roles as caregivers were, whether they had had the main caring responsibilities, whether or not they had a family of their own, and whether they had a job.

We found that the informants’ dominant desires and strategies in the coping efforts were gained by developing *detachment*. The detachment process includes the following main approaches: situational changes, cognitive strivings, and emotional reactions. These approaches are related to circumstances, events, and the progression of the disease. The informants sometimes had one dominant approach, but at the times when they used multiple strategies, the strategies were often interrelated at different stages, influencing and modifying each other. Coping efforts took place while the young person was maturing and, as the parent's dementia progressed, the situation characteristically changed. The outcome was the result of a mixture of actions taken by the informants (such as moving away), changes in the parent's and the family's situations, and cognitive and emotional adaptations. The main trend in these changes and coping efforts was *becoming more detached* and finding more space and greater independence for their own development: becoming an *I* by being more in control of their own lives, being able to develop more according to their own needs and interests, and prioritizing themselves.

*Moving apart* was the outcome of the parent moving into an NH, attending a day care programme, or being in respite care for a period. It may also have been a result of the adult children's decision to leave the parental home either gradually or abruptly and temporarily or permanently. The movement toward *greater personal distance* was influenced by greater physical distance, the informant's gradual acceptance of the diagnosis and its consequences, loss of personal contact because of the disintegration of the parent's personality, and cognitive (re)assessments of the parent–child relation (Lazarus & Folkman, 1984). *Calmer emotional reactions* were also reported by the informants. This subcategory refers to the point where the informant's emotions become less intense. The anger became subdued, and the initial shock of the diagnosis was overcome. The emotional spectrum was still great, but the content of the feelings was now more mellow; it was now more sorrow and sadness than shock.

Part of the detachment process is greater *avoidance* of intense involvement with the parent. A legacy of the psychoanalytical tradition, avoidance has mostly been considered dysfunctional, even unhealthy, and a deterrent from more constructive mastering of the stressful problems. Avoidance has mainly been conceived of as a sort of self-deception, a denial or wishful thinking, or an escape from active coping with the realities. Referring to a study by Connor-Smith, Compas, Wadsworth, Thomsen, and Saltzman (2000) that focused on coping in adolescence in general, some researchers who observe adolescent children of parents with YOD argue in the same vein. The authors of one study stated that better adjustment is associated with less reliance on avoidant coping (Millenaar et al., 2014). Similarly, a number of other researchers have stated that the advice given to adolescents/young adult children of parents with YOD, as well as to older caregivers—to reserve more time for themselves and participate in activities outside the family—can be seen as a sort of encouragement of unhealthy avoidance (Bruvik, Ulstein, Ranhoff, & Engedal, 2012; Gelman & Greer, 2011). However, coping theories (Lazarus & Folkman, 1984) and theories on successful ageing (Baltes & Baltes, 1990; Baltes & Carstensen, 1996) have emphasized that avoidance can be positive when control of the situation is not possible (Heckhausen & Schulz, 1995; Heckhausen, Wrosch, & Schulz, 2010).

We found that avoidance, as part of the detachment process, can be adaptive or necessary for these informants for some problems and at some moments. It can be a protective factor. The individuals can portion out what they are able to consider and cope with at the time. As the study shows, the informants can gradually come to a more realistic acceptance of the dementia and its implications.

The situation of children growing up with parents with YOD differs in significant ways from that of children growing up with parents with other chronic and serious diseases, such as cancer (Thastum, Johansen, Gubba, Olesen, & Romer, 2008), HIV (Keigher, Zabler, Robinson, Fernandez, & Stevens, 2005), and psychiatric diseases (Beardslee, Verslag, & Gladstone, 1998). Some of these disorders may already be well known to others in the children's lives. Therefore, conditions such as cancer can be easier to talk about. The serious diseases affecting parents vary according to whether they are progressive or have an outcome that gives hope. They differ depending on the parents’ physical functioning, mental capacities, personalities, and reaction patterns, as well as on whether the diseases are well known to the public or are stigmatized (Hutchinson et al., 2014). They also differ based on whether they are normative or non-normative (Gelman & Greer, 2011), usual or unusual, and on-time or off-time in the life course of both parent and child. In these respects, YOD is unique, and growing up as a child of a parent with YOD is exceptional.

Thus, we found that the adult children used combinations of strategies—action-oriented, cognitive, and emotional—that we, on a higher analytical level, have summarized as more detachment from their parent with dementia. The strategies were adapted to the circumstances, changing over time. Maturing was part of the picture. This detachment may provide protection from some of the negative influences of a very stressful upbringing.

This study has shown that, through greater detachment, the informants developed more resilience as they better mastered the challenges, a more robust capacity to handle adversity, and a greater ability to accept and adapt to the situation. We suggest that the later lives of these adult children may not be as problematic as the circumstances might indicate.

Studies have demonstrated that children of parents with YOD feel overlooked as individuals and their needs have been neglected (Barca et al., 2014; Johannessen et al., 2015). The children have to be seen and get recognition and support. The support systems for families of persons with YOD should be family-oriented (Gelman & Greer, 2011; Haugen, 2012; Skovdal et al., 2007), but at the same time individuals who support these families should recognize that all family members have unique personal experiences regarding how dementia has influenced their lives. For people in “emerging adulthood” (Arnett, 2004), support systems are vital to help them establish themselves as individuals outside the family, gain recognition, maintain friendships, discover new arenas, and get on with their own lives.

### Strengths and limitations

With strategic sampling of a heterogeneous group of young adult children of parents with YOD, we have captured these young adults’ varied experiences and life situations and the complexities of human life (Corbin & Strauss, 2008, p. 305). Hopefully, the participants will find a “fit” between their experiences and the research accounts, what Charmaz (2006, p. 182–185) called “resonance.” We have emphasized procedures to establish trustworthiness (Lincoln & Guba, 1990).

However, to further explore the strategies that such adult children use and the fruitfulness of the concept of detachment, other studies in other contexts are necessary. Confirmation by multiple comparison groups will strengthen the evidence and specify the circumstances (Corbin & Strauss, 2008). Limitations of the study include the rather short time period between the interview phases and the small sample size. Future researchers should engage with participants over a longer period of time in order to capture the further development of coping and resilience in their subjects.

## Conclusions

The main finding in this study is that almost all the informants felt that their life situation had improved and that they coped with the situation with their parent in several ways. They used various coping strategies. Sometimes one dominated, but usually several were combined. Moreover, the direction was usually greater detachment. Resilience gradually evolved as the informants mastered the situation over time. It is vital for the personal development of adult children to gain a sense of identity, achieve self-reliance, increase coping capacities, mature as (looked at) individuals, and feel supported socially. Public services must respond to the adult children's needs for individual development and support the children's coping capacities.
